# A System of Surgery; Pathological, Diagnostic, Therapeutic, and Operative

**Published:** 1864-10

**Authors:** 


A System of Surgery; Pathological, Diagnostic, Therapeutic, and Operative. By Samuel D. Gross, M.D., Prof. of Surgery in Jefferson Medical College of Philadelphia; Surgeon to the Philadelphia Hospital; Member of the Imperial Royal Medical Society of Vienna, &c., &c. Illustrated by over thirteen hundred engravings. Third Edition, much enlarged and revised; in two volumes. Philadelphia: Blanchard & Lea. 1864.

The first edition of this great and valuable work on surgery, was noticed at length in this Journal, at the time it was issued from the press. Since that time it has become so well known to the profession, both in this country and Europe, that it needs no further commendation from us. The present edition has been carefully revised by the author. It consists of two volumes of more than 1000 pages each; printed on fair type, and elegantly bound. It is probably the most complete and valuable treatise on surgery, accessible to American students and practitioners.

For sale by S. C. Griggs & Co., Lake Street, Chicago.

